# Low-dose celecoxib-loaded PCL fibers reverse intervertebral disc degeneration by up-regulating CHSY3 expression

**DOI:** 10.1186/s12951-023-01823-4

**Published:** 2023-03-03

**Authors:** Yunhao Wang, Genjiang Zheng, Xiaoxing Xie, Wei Yu, Jianxi Wang, Fazhi Zang, Chen Yang, Qiangqiang Xiao, Rongcheng Zhang, Leixin Wei, Xiaodong Wu, Lei Liang, Peng Cao, Chen Xu, Jing Li, Bo Hu, Tao Zhang, Jinglei Wu, Huajiang Chen

**Affiliations:** 1 Spine Center, Department of Orthopedics, Changzheng Hospital, Naval Medical University, Shanghai, 200003 China; 2 Shanghai Engineering Research Center of Nano-Biomaterials and Regenerative Medicine, College of Biological Science and Medical Engineering, Donghua University, Shanghai, 201620 China; 3 Engineering Research Center of Cell & Therapeutic Antibody, Ministry of Education, and School of Pharmacy, Shanghai Jiao Tong University, Shanghai, 200240 China; 4 Department of Orthopaedics, Shanghai Jiao Tong University Affiliated Sixth People’s Hospital, Shanghai, 200233 China; 5 Department of Bioinformatics, Center for Translational Medicine, Naval Medical University, Shanghai, 200433 China

**Keywords:** Intervertebral disc degeneration, Low-dose celecoxib, PGE2, CHSY3

## Abstract

Intervertebral disc degeneration (IDD) has been identified as one of the predominant factors leading to persistent low back pain and disability in middle-aged and elderly people. Dysregulation of Prostaglandin E2 (PGE2) can cause IDD, while low-dose celecoxib can maintain PGE2 at the physiological level and activate the skeletal interoception. Here, as nano fibers have been extensively used in the treatment of IDD, novel polycaprolactone (PCL) nano fibers loaded with low-dose celecoxib were fabricated for IDD treatment. In vitro studies demonstrated that the nano fibers had the ability of releasing low-dose celecoxib slowly and sustainably and maintain PGE2. Meanwhile, in a puncture-induced rabbit IDD model, the nano fibers reversed IDD. Furthermore, low-dose celecoxib released from the nano fibers was firstly proved to promote CHSY3 expression. In a lumbar spine instability-induced mouse IDD model, low-dose celecoxib inhibited IDD in CHSY3^wt^ mice rather than CHSY3^−/−^ mice. This model indicated that CHSY3 was indispensable for low-dose celecoxib to alleviate IDD. In conclusion, this study developed a novel low-dose celecoxib-loaded PCL nano fibers to reverse IDD by maintaining PGE2 at the physiological level and promoting CHSY3 expression.

**Supplementary Information:**

The online version contains supplementary material available at 10.1186/s12951-023-01823-4.

## Introduction

Intervertebral disc degeneration (IDD) has been identified as one of the predominant factors causing persistent low back pain and disability in middle-aged and elderly people [1]. Breakdown and abnormal synthesis of extracellular matrix (ECM) paly essential roles in physiology and pathological process [2]. Dysregulated ECM metabolism leads to a decrease of hydration in NP, reduction of intervertebral space height, and the declined capacity of the spine to withstand external mechanical force [3]. Thus, we aim to alleviate IDD by regulating the homeostasis of ECM.

Prostaglandin E2 (PGE2) can regulate ECM-related processes such as cell proliferation, migration, and extracellular matrix synthesis and degradation. For example, PGE2 has been shown to stimulate the production of matrix metalloproteinases, which are enzymes that break down ECM components, as well as promote the synthesis of various ECM components, including proteoglycans and collagens [4]. These changes to the ECM can contribute to skeletal system homeostasis and intervertebral disc degeneration [5–7]. PGE2 at a physiological level is helpful to activate skeletal interoception to regulate skeletal system homeostasis, whereas higher PGE2 concentrations induced pain and did not activate skeletal interoception [7]. Similarly, PGE2 at physiological levels may also regulate intervertebral disc degeneration through the process of interoception.

Celecoxib, a selective inhibitor of COX2, is currently recommended as a drug of choice for treating low back pain caused by IDD [8]. Celecoxib regulates PGE2 to mediate skeletal interoception and improve IDD [7]. The presence of celecoxib normalizes PGE2 production and ECM homeostasis [9]. However, high-dose celecoxib has been reported to aggravate the degradation of ECM [10, 11]. This may partly be because celecoxib inhibits the release of PGE2 in a dose-dependent manner, and low-dose celecoxib can maintain PGE2 at a physiological level to regulate the skeletal interoception [7, 12]. Peng et al. reported that 20 mg kg^−1^ orally per day decreased spinal pain hypersensitivity and maintained PGE2 at a physiological level [7]. Herein, we considered a dose that keeps PGE2 at physiological level was a low dose for the topical application of celecoxib.

Recently, nano fibers have been reported for improving matrix infiltration micromechanical properties, and are increasingly recognized to play an essential role in therapeutic applications of IDD [13, 14]. Due to membranes’ property of high porosity and large surface area to volume ratio, they are suitable to be carriers for drug delivery [15, 16]. The membranes ensure long-term and stable drug release [17, 18]. Besides, they can avoid repeated local drug injections and gastrointestinal symptoms of some drugs such as inhibitors of COX2 [19, 20]. Celecoxib-loaded nano fibers were reported to reduce the levels of inflammatory mediators [21]. However, high-dose celecoxib was preferred in previously reported nano fibers. Researchers had identified the importance of low-dose celecoxib in maintaining PGE2, but nano fibers which had the steady continuous release of low-dose celecoxib were not yet available. Based on the fact that low-dose celecoxib can maintain PGE2 in the physiological level, we managed the first synthesis of the PCL nanofiber which could continuously release low-dose celecoxib.

In this study, we firstly fabricated low-dose celecoxib-loaded PCL nano fibers to release celecoxib slowly and sustainably. We then established an IDD rabbit model and a lumbar spine instability (LSI) mouse model. In the IDD rabbit model, the nano fibers were proven to improve ECM anabolic metabolism, regulate skeletal interoception activity, and reverse degeneration. In the LSI mouse model, CHSY3 was confirmed to be indispensable in the low-dose celecoxib treatment for IDD. To summarize, we indicated the mechanism of IDD and discovered a novel approach to inhibit IDD.

## Results

### Characterization of celecoxib-loaded PCL nano fibers

**Fig. 1:**
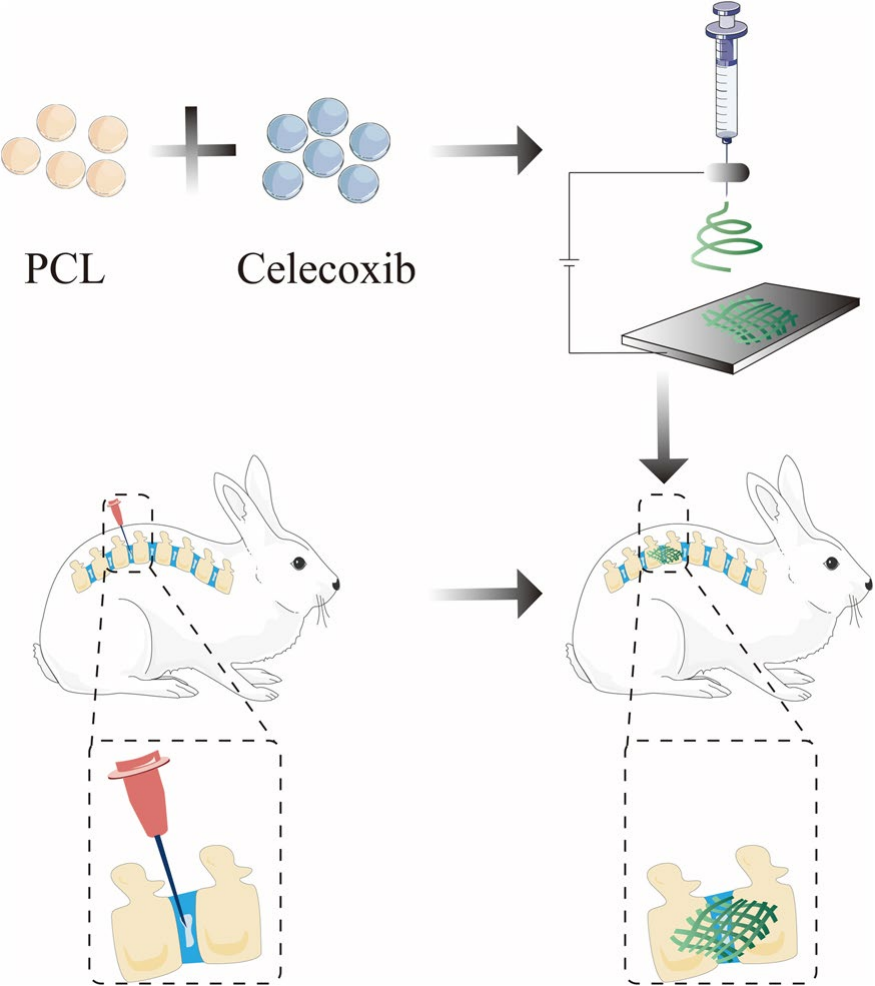
Schematic of the processes for fabrication of PCL-loaded celecoxib nano fibers and its treatment for IDD in vivo

The celecoxib-loaded PCL nano fibers were fabricated by electrospinning and evaluated for their application in intervertebral disc tissue engineering (Fig. 1).

**Fig. 2:**
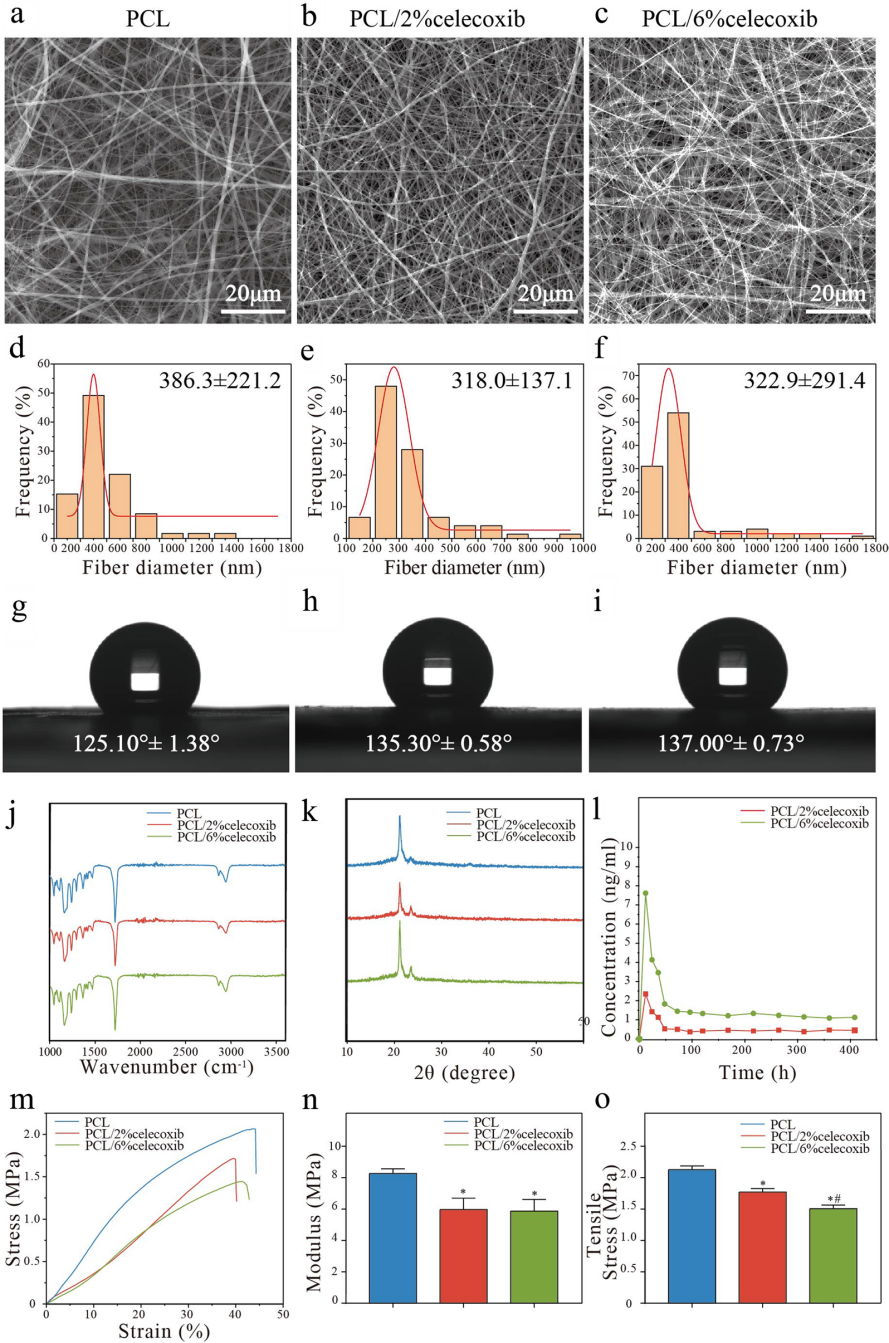
Representative **a**–**c** SEM, **d**–**f** diameter distribution, and **g**–**i** contact angles of PCL, PCL/2% celecoxib, and PCL/6% celecoxib nano fibers. **j**, **k** FTIR (**j**) and XRD (**k**) of PCL, PCL/2% celecoxib, and PCL/6% celecoxib nano fibers. **l** Release percentage of PCL, PCL/2% celecoxib, and PCL/6% celecoxib nano fibers. **m**–**o** Mechanical properties of PCL, PCL/2% celecoxib, and PCL/6% celecoxib nano fibers. Values are presented as means ± SD, *P < 0.05 compared with the PCL group, ^#^P < 0.05 compared with the PCL/2% celecoxib group, and ^†^P < 0.05 compared with the PCL/6% celecoxib group

As shown in Fig. 2a–c, the nano fibers form a porous system with relatively uniform fibers and a smooth surface with no visible drug crystals. The fiber diameter distribution plots demonstrated that the average diameters of PCL, PCL/2% celecoxib fibers, and PCL/6% celecoxib fibers were 386.3 ± 221.2 nm, 318.0 ± 137.1 nm, and 322.9 ± 291.4 nm, respectively (Fig. 2d–f). To find out the hydrophobic effects of celecoxib on the surface properties of PCL nano fibers, we took water contact angle measures. The water contact angles for PCL, PCL/2% celecoxib, and PCL/6% celecoxib fibers were 124.10 ± 1.38°, 135.30 ± 0.58°, and 137.00 ± 0.73°, respectively (Fig. 2g–i). The water contact angles were more than 135° for all celecoxib-loaded nano fibers, which indicated the hydrophilic property of materials. The addition of lipophilic drug celecoxib helped to increase the hydrophobicity of PCL nano fibers because celecoxib was rich in hydrophobic groups.

To detect the composition of celecoxib-loaded PCL nano fibers, we performed Fourier-transform infrared spectroscopy (FTIR) and X-ray diffraction (XRD). The FTIR analysis indicated that absorption peaks were at 2941, 2868 (vs; ν(C–H)), 1720 (s; ν(C=O)), and 1157 cm^−1^ (s; ν(C–O)), and those were the characteristics of PCL (Fig. 2j) [22]. The XRD analysis showed that the two dominating peaks of PCL appeared at 21.4° and 23.8° (Fig. 2k) [22]. From the FTIR and XRD characteristic peaks of pristine PCL, no obvious changes were found, confirming that the chemical structures of celecoxib and PCL remained intact after electrospinning.

The cumulative release of celecoxib from celecoxib-loaded nano fibers was investigated by incubation in the release medium. It took about five days (PCL/6% celecoxib fibers) and more than 1 week (PCL/2% celecoxib fibers) for the nano fibers to release approximately 80% of the loaded drug with a burst release of about 50% of the drug in the first 2 days (Additional file 1: Fig. S1a). As shown in Fig. 2l, celecoxib showed burst release at the first 96 h, releasing 62.92% and 78.32% of the drugs, followed by controlled and sustained release in the next 312 h. At the end of 17 days, celecoxib was almost completely released Cumulative amounts of celecoxib were calculated based on different numbers of electrospun fibers in the membrane, and the release date will serve as a guide for the design of animal experiments.

To evaluate the effect of celecoxib components on the mechanical properties of PCL/celecoxib nano fibers, we performed stress–strain measurements. As shown in Fig. 2m–o, the Young’s moduli of PCL, PCL/2% celecoxib, and PCL/6% celecoxib nano fibers were 8.28 ± 0.17 MPa, 5.97 ± 0.42 MPa, and 5.868 ± 0.43 MPa, respectively. The tensile strengths were 2.12 ± 0.04 MPa, 1.77 ± 0.03 MPa, and 1.51 ± 0.03 MPa, respectively.

### Celecoxib-loaded PCL nano fibers inhibiting IL-1β-induced degeneration of NP in vitro

**Fig. 3:**
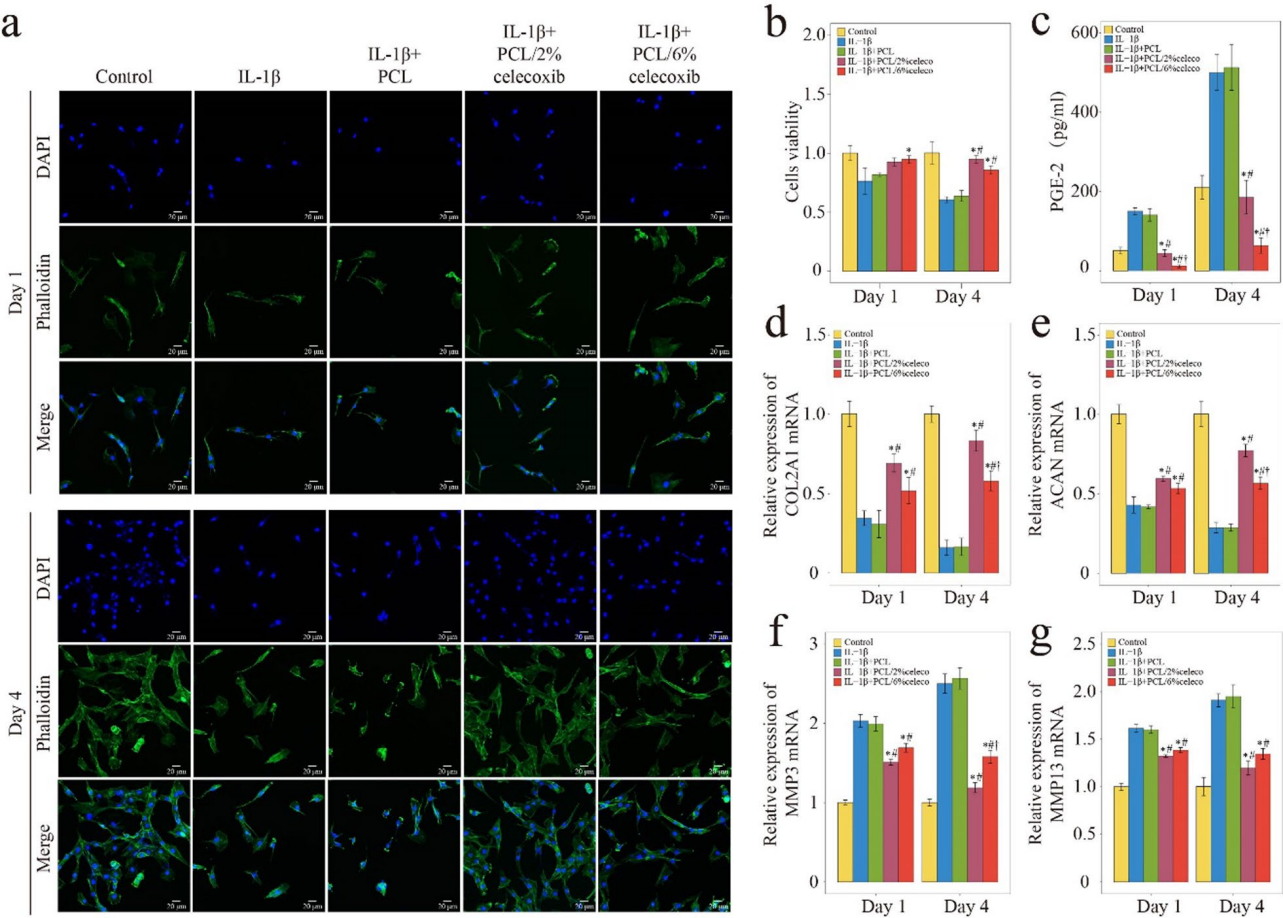
The viability, PGE-2 release, and ECM synthesis of NP cells on PCL, PCL/2% celecoxib, and PCL/6% celecoxib nano fibers. **a**, **b** The results of phalloidin analysis after NP cells were cultured for 1 day and 4 days. **c** PGE2 release of NP cells detected by ELISA. **d**–**g** The results of Col2A1 (**d**), ACAN (**e**), MMP3 (**f**), and MMP13 (**g**) expression were detected by PCR. *P < 0.05 compared with the IL-1β group, ^#^P < 0.05 compared with the IL-1β + PCL celecoxib group, and ^†^P < 0.05 compared with the IL-1β + PCL/2% celecoxib group

Inflammatory cytokines, especially IL-1β, are important mediators of IDD and regulators of NP cell activity [23]. To investigate whether celecoxib-loaded PCL nano fiberscan promote the activity of NP cells in an inflammatory microenvironment, we cultured NP cells on PCL/celecoxib fibrous nano fibers and treated them with IL-1β-supplemented medium for 1 day and 4 days. As shown in Fig. 3a and b, the number of NP cells gradually increased over time, the control group, the IL-1β + PCL/2% celecoxib group, and the IL-1β + PCL/6% celecoxib group NP cells grew well, and NP cells grew poorly in the IL-1β group and the IL-1β + PCL group. Notably, cell viability increased with celecoxib, most significantly with 2% celecoxib. This result suggests that PCL/2% celecoxib nano fibers have different mechanisms for regulating NP cell activity compared with PCL/6% celecoxib nano fibers.

In the ELISA assay, PGE2 release increased significantly in IL-1β-induced NP cell supernatant medium and was effectively alleviated after the treatment of celecoxib-loaded PCL nano fibers (Fig. 3c). Additionally, PGE2 concentrations were similar in the control and the PCL/2% celecoxib nano fiber groups. Thus, PCL/2% celecoxib nano fibers were proven to have the effect of maintaining the physiological concentration of PGE2, and the dose of celecoxib in PCL/2% celecoxib nano fibers was considered to be a low dose according to previous researches [7]. In contrast, the dose of celecoxib in PCL/6% celecoxib electrospun nano fibers was regarded to be a high dose.

Previous studies demonstrated that Collagen II (Col II) and Aggrecan (ACAN) were the two important constituents of ECM and degenerative NP cells had a decreased expression of Col II and ACAN [31]. On the other hand, matrix metalloprotein 3 (MMP3) and matrix metalloprotein 13 (MMP13) were highly expressed in degenerative NP cells [32]. The PCR result showed that after IL-1β induced NP cells, the relative expressions of ACAN and Col II mRNA were significantly decreased, and the expressions of MMP3 and MMP13 were significantly increased (Fig. 3d–g). PCL/celecoxib nano fibers, especially PCL/2% celecoxib nano fibers, effectively promoted Col II and ACAN expression and inhibited MMP3 and MMP13 expression. The better effect of PCL/2% celecoxib nano fibers indicated that low-dose celecoxib was superior to high-dose celecoxib in promoting ECM anabolism and inhibiting ECM catabolism.

Celecoxib-loaded PCL nano fibers significantly suppressed NP cells’ viability, reduced PGE2 release, and promoted ECM synthesis. Reversely, PCL/2% celecoxib nano fibers increased viability, promoted ECM anabolism, and inhibited ECM catabolism of NP cells than PCL/6% celecoxib nano fibers. Meanwhile, PCL/2% celecoxib nano fibers maintained PGE2 in physiological concentrations, but PCL/6% celecoxib nano fibers kept PGE2 at very low concentration levels. These results suggested that PCL/2% celecoxib nano fibers regulated viability and ECM synthesis by maintaining PGE2 in physiological concentrations.

### Celecoxib-loaded PCL nano fibers alleviating IDD in vivo

**Fig. 4:**
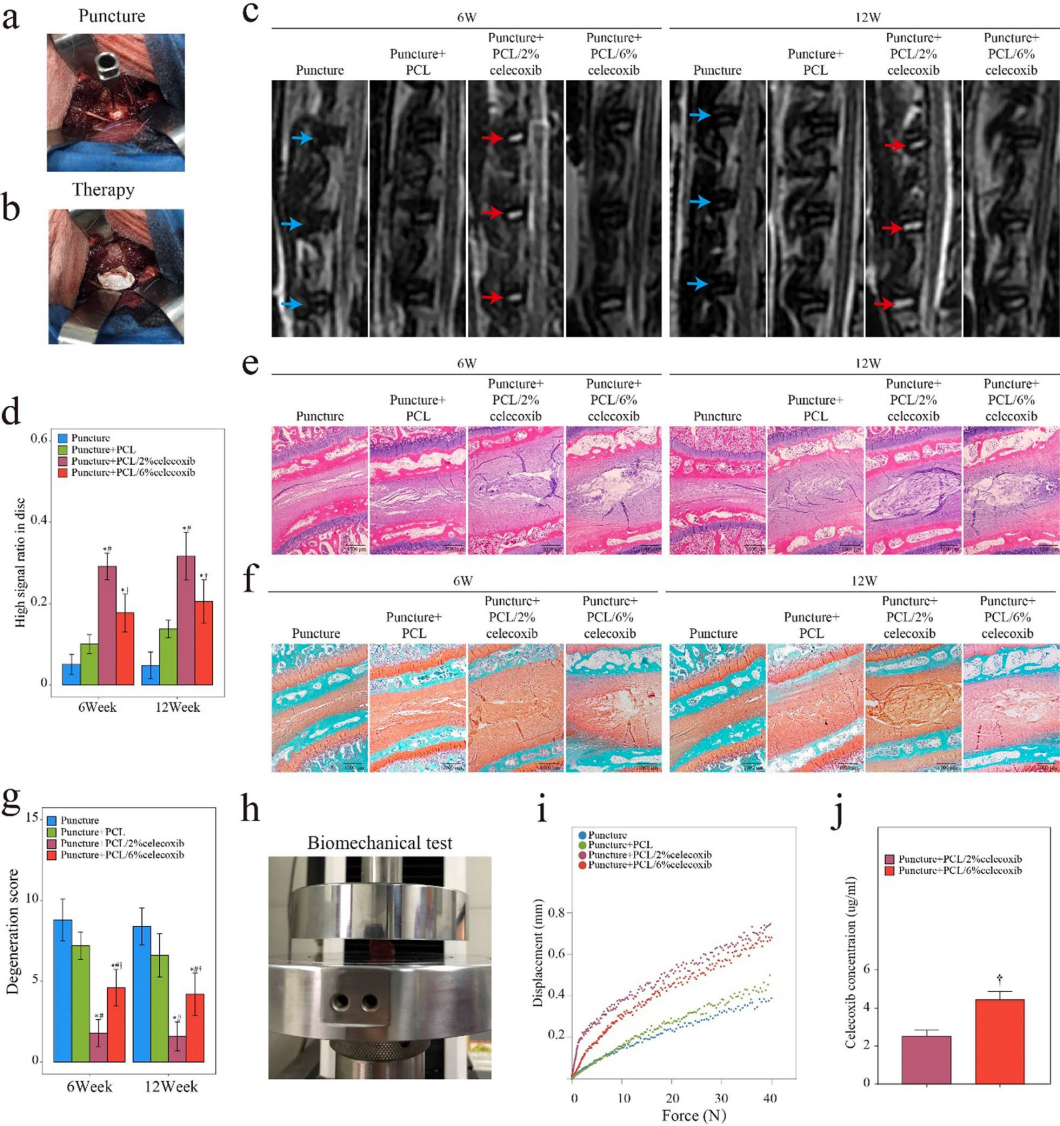
Evaluation of IDD therapy of PCL, PCL/2% celecoxib, and PCL/6% celecoxib nano fibers in vivo. **a**, **b** IDD rabbit puncture model and the therapy with nano fibers. **c** MRI of rabbit intervertebral discs treated with PCL, PCL/2% celecoxib, and PCL/6% celecoxib nano fibers. Blue arrow indicating loss of hydration and the red arrow indicating recovery of hydration. **e** HE staining, and **f** SOFG staining of rabbit intervertebral disc treated with PCL, PCL/2% celecoxib, and PCL/6% celecoxib nano fibers. **d** High signal ratio in the disc of MRI. **g** Degeneration score of HE. **h**–**i** Biomechanical properties of the intervertebral disc. **j** Celecoxib concentrations in the intervertebral disc. *P < 0.05 compared with the puncture group, ^#^P < 0.05 compared with the puncture + PCL celecoxib group, and ^†^P < 0.05 compared with the puncture + PCL/2% celecoxib group

To evaluate PCL/celecoxib nano fibers’ ability to alleviate IDD, we performed a 12-week in vivo experiment in rabbits. A typical IDD model induced by needle punctures was established, and the breach was followed by the implantation of celecoxib-loaded PCL nano fibers (Fig. 4a, b). Based on the treatment, rabbits were divided into four groups: a puncture group, a puncture with PCL nano fiber group, a puncture with PCL/2% celecoxib nano fiber group, and a puncture with PCL/6% celecoxib nano fiber group. All rabbits survived the operation and recovered well without postoperative complications.

#### Celecoxib-loaded PCL nano fibers promoting NP hydration

MRI was performed in all rabbits at 6 weeks and 12 weeks after the operation to detect NP hydration. T2-weighted images were used to assess the water content of NP, and a higher signal ratio indicated a higher degree of hydration [24]. The T2-weighted images at 6 weeks and 12 weeks after surgery (Fig. 4c) showed very low hydration in the puncture group and PCL nano fiber group, but compared with the puncture group, hydration remarkably increased in the PCL/2% celecoxib nano fiber group and the PCL/6% celecoxib nano fiber group. Interestingly, the red arrows indicated that the PCL/2% celecoxib nano fiber group had the highest hydration among all the groups. The statistical graph of the ratio of the high signal also showed that higher and brighter signals were detected in the NP of the PCL/2% celecoxib nano fiber group (Fig. 4c). The sharp decline in collagens and proteoglycans resulted in the loss of hydration and further IDD [25]. These results suggested that low-dose celecoxib-loaded PCL nano fibers could better promote NP hydration and alleviate IDD.

#### Celecoxib-loaded PCL nano fibers decreasing the degeneration score

HE and SO staining demarcated rabbit discs’ morphological characteristics, and histological scores were graded according to HE results (Fig. 4e–g). In the puncture group, massive loss of proteoglycans and obvious tears of the annulus fibrosus appeared. In the PCL nano fiber group, despite the loss of proteoglycans, the disc height was obviously restored, and the annulus fibrosus was relatively ordered. In the PCL/6% celecoxib nano fiber group, proteoglycans were partly restored, and the annulus fibrosus was relatively intact. In the PCL/2% celecoxib nano fiber group, NP was rich in proteoglycans, and annulus fibrosus structures appeared orderly. According to a new histological classification, a higher score indicated a greater degree of degeneration of intervertebral discs [26]. As shown in Fig. 4g, the degeneration scores in the puncture group were significantly higher than the other groups, and the degeneration scores were effectively reduced in the PCL/2% celecoxib nano fiber group and the PCL/6% celecoxib nano fiber group. Importantly, the PCL/2% celecoxib nano fiber group had the lowest degeneration scores. Thus, it could be concluded that low-dose celecoxib-loaded PCL nano fibers were more effective in improving morphological characteristics and delaying the degeneration process.

#### Celecoxib-loaded PCL nano fibers enhancing biomechanical function and measures of celecoxib concentration

During IDD, the biomechanical function of the intervertebral disc turns to progressive stiffening and remodeling [27]. To evaluate the biomechanical function of celecoxib-loaded PCL nano fibers in therapy, we performed a biomechanical test at the end of the 12-week experimental period (Fig. 4h). As shown in Fig. 4i, the biomechanical properties of the intervertebral disc were displayed in the representative force displacement and creep curves. The puncture group and the PCL nano fiber group had minimal displacements under low pressure, suggesting that their biomechanical ability to withstand load pressure decreased. The displacements of the PCL/2% celecoxib nano fiber group and the PCL/6% celecoxib nano fiber group were much higher than those of the puncture group, and the creep curve of the PCL/2% celecoxib nano fiber group showed a steeper slope. These results indicated that low-dose celecoxib-loaded PCL nano fibers could better enhance the biomechanical function of promoting segmental mobility and biomechanical ability to bear loading pressure on the intervertebral disc.

PCL/2% celecoxib nano fiber did particularly well for IDD and we further investigated the concentration of celecoxib by LC–MS/MS. Figure 4j revealed that 2232 ng/mL was detected in the PCL/2% celecoxib nano fiber and this concentration was a low dose in the intervertebral disc. In contrast, the concentration of high-dose celecoxib in the intervertebral disc was 4353 ng/mL. Therefore, we could define the low-dose celecoxib based on the concentration of celecoxib in the intervertebral disc, which was about 2232 ng/mL.

#### Celecoxib-loaded PCL nano fibers relieving pain states and regulating interoception

**Fig. 5:**
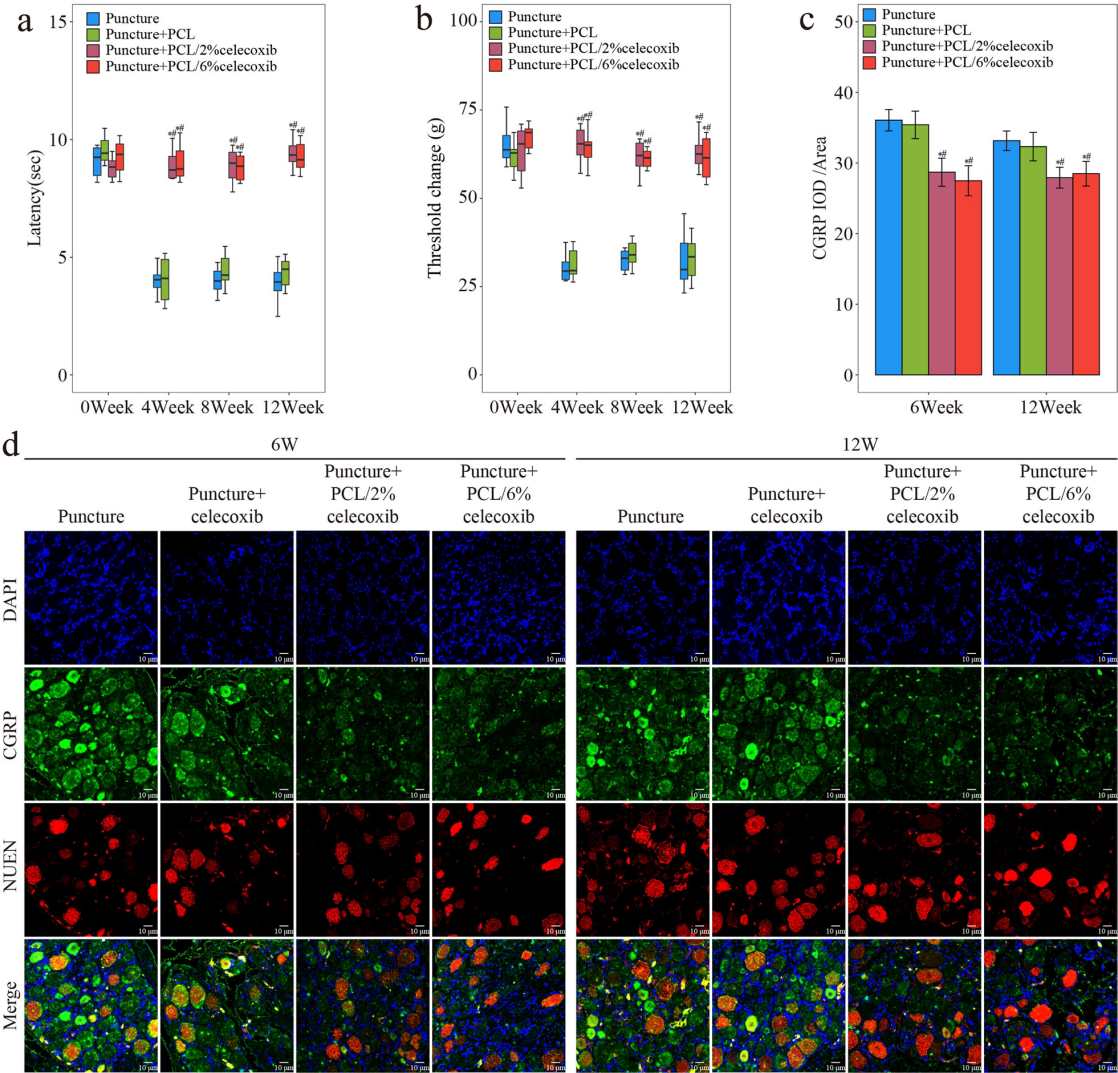
Evaluation of pain condition with the treatment of PCL, PCL/2% celecoxib nano fibers, and PCL/6% celecoxib nano fibers. **a** Hot plate test and **b** Von Frey test of rabbits treated with PCL, PCL/2% celecoxib nano fibers, and PCL/6% celecoxib nano fibers for 0 weeks, 4 weeks, 8 weeks, and 12 weeks. **c**–**d** IHC analysis of CGRP, NUEN, and DAPI of DRG of rabbit treated with PCL, PCL/2% celecoxib nano fibers, and PCL/6% celecoxib nano fibers for 6 weeks and 12 weeks. *P < 0.05 compared with the puncture group, ^#^P < 0.05 compared with the puncture + PCL celecoxib group, and ^†^P < 0.05 compared with the puncture + PCL/2% celecoxib group

To evaluate the rabbits’ neuropathic pain condition, we performed the Von Frey test and a hot plate test at week 4, week 8, and week 12 after operation [28]. As shown in Fig. 5a and b, there were no obvious differences between the four groups preoperatively. In the puncture group, mechanical punctate withdrawal threshold values and hot plate test latencies were reduced significantly at week 4, week 8, and week 12 after the operation. In the PCL group, threshold values and latencies increased to a certain extent but there was no significant difference with the puncture group. On the other hand, in the PCL/2% celecoxib nano fiber group and the PCL/6% celecoxib nano fiber group, postoperative threshold values and latencies appeared time-dependent progressive increase, and there was no difference between the two groups.

CGRP was one of the most important functional neuropeptides secreted by sensory nerve and was reported to promote CS content to stimulated NP cells [29]. Research suggested that CGRP may contribute to IDD by sensitizing the sensory neurons that are responsible for detecting and transmitting signals from the intervertebral discs. This may lead to an increase in pain and other symptoms as the degeneration progresses [30, 31]. Meanwhile, the COX2 inhibitor mitigated pain by suppressing CGRP release [32]. After 6 weeks and 12 weeks postoperatively, the expression of CGRP was significantly higher in the puncture group and the PCL nano fiber group, but the increase of CGRP was abolished in the PCL/2% celecoxib nano fiber group and the PCL/6% celecoxib nano fiber group (Fig. 5c, d). In addition, no statistical difference was found in CGRP expression between the PCL/2% celecoxib nano fiber group and the PCL/6% celecoxib nano fiber group. Thus, low-dose celecoxib-loaded PCL nano fibers and PCL/high-dose celecoxib nano fibers had good pain-relieving effects. These findings showed that the levels of CGRP changed in response to IDD and its inhibition by celecoxib-loaded PCL nano fibers. CGRP, secreted by sensory nerves, was proved to be an important regulatory factor in IDD in our previous research [29]. In conclusion celecoxib-loaded PCL nano fibers may regulate IDD through interoception by modulating the levels of CGRP.

#### Celecoxib-loaded PCL nano fibers regulating NP ECM metabolism

**Fig. 6:**
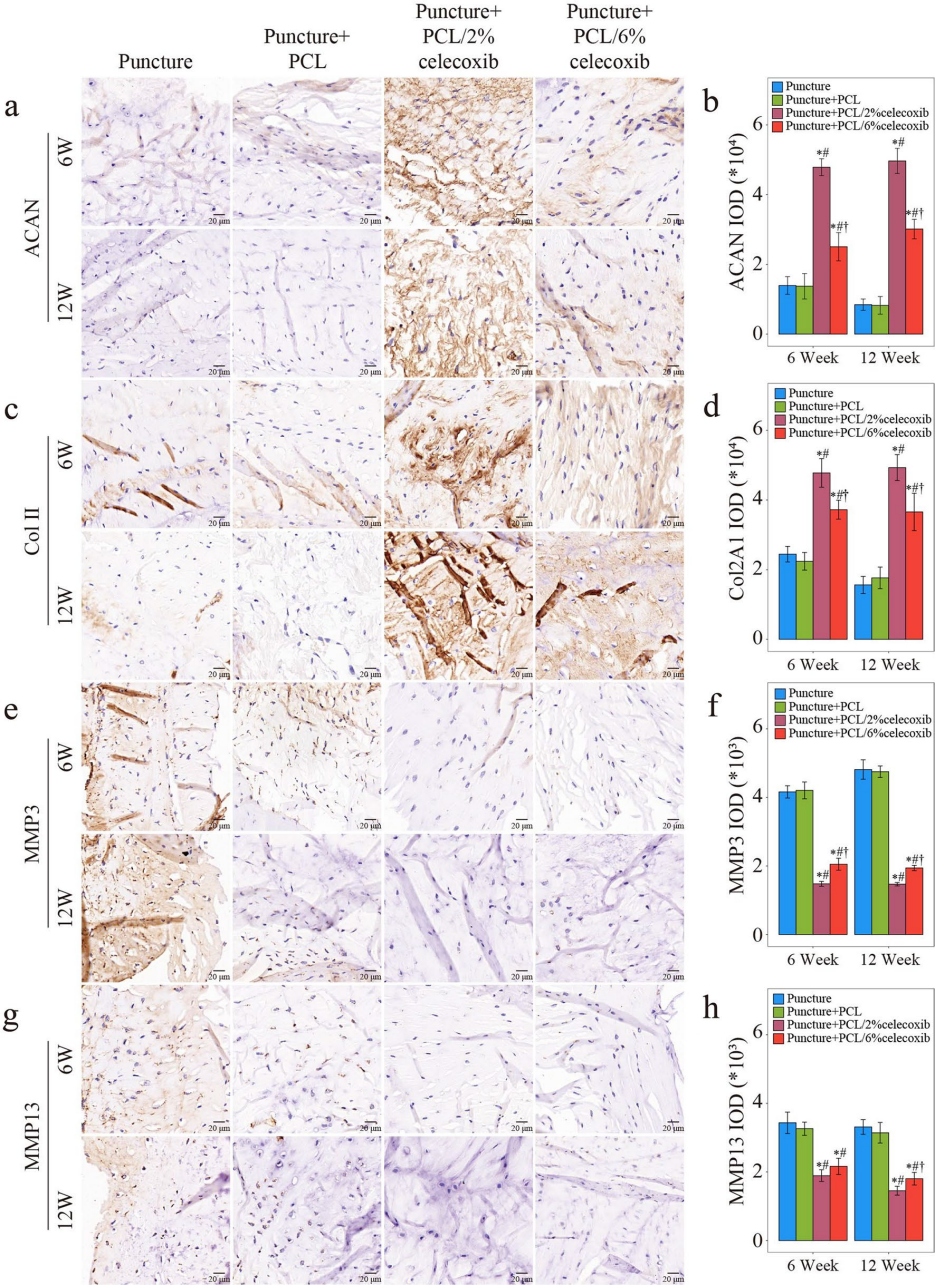
Evaluation of NP ECM metabolism with the treatment of PCL, PCL/2% celecoxib nano fibers, and PCL/6% celecoxib nano fibers. **a**–**h** IHC of ACAN (**a**, **b**), Col2A1 (**c**, **d**), MMP3 (**e**, **f**), and MMP13 (**g**, **h**) in NP of rabbits treated with PCL, PCL/2% celecoxib nano fibers, and PCL/6% celecoxib nano fibers for 6 weeks and 12 weeks. *P < 0.05 compared with the puncture group, ^#^P < 0.05 compared with the puncture + PCL celecoxib group, and ^†^P < 0.05 compared with the puncture + PCL/2% celecoxib group

To evaluate NP’s ECM synthesis and breakdown, we conducted IHC in rabbit intervertebral discs at week 6 and week 12 after surgery. As shown in Fig. 6a–d, a small amount of ACAN and Col II was expressed in the puncture group and the PCL group. Meanwhile, ACAN and Col II were both highly expressed in the PCL/2% celecoxib nano fiber group and the PCL/6% celecoxib nano fiber group. On the contrary, the expression levels of MMP3 and MMP13 were higher in the puncture group and the PCL group. At the same time, the Sham group, the PCL/celecoxib group, the PCL/2% celecoxib nano fiber group, and the PCL/6% celecoxib nano fiber group, had a small amount of expression of MMP3 and MMP13 (Fig. 6e–h). It is worth noting that the PCL/2% celecoxib nano fiber group had the highest expression of ACAN and Col II and the lowest expression of MMP3 and MMP13. These results suggested that celecoxib-loaded PCL nano fibers had the effect of promoting ECM anabolism and inhibiting ECM catabolism, and were more effective in regulating NP ECM metabolism.

#### Celecoxib-loaded PCL nano fibers inhibiting the expression of inflammatory cytokines and COX2

**Fig. 7:**
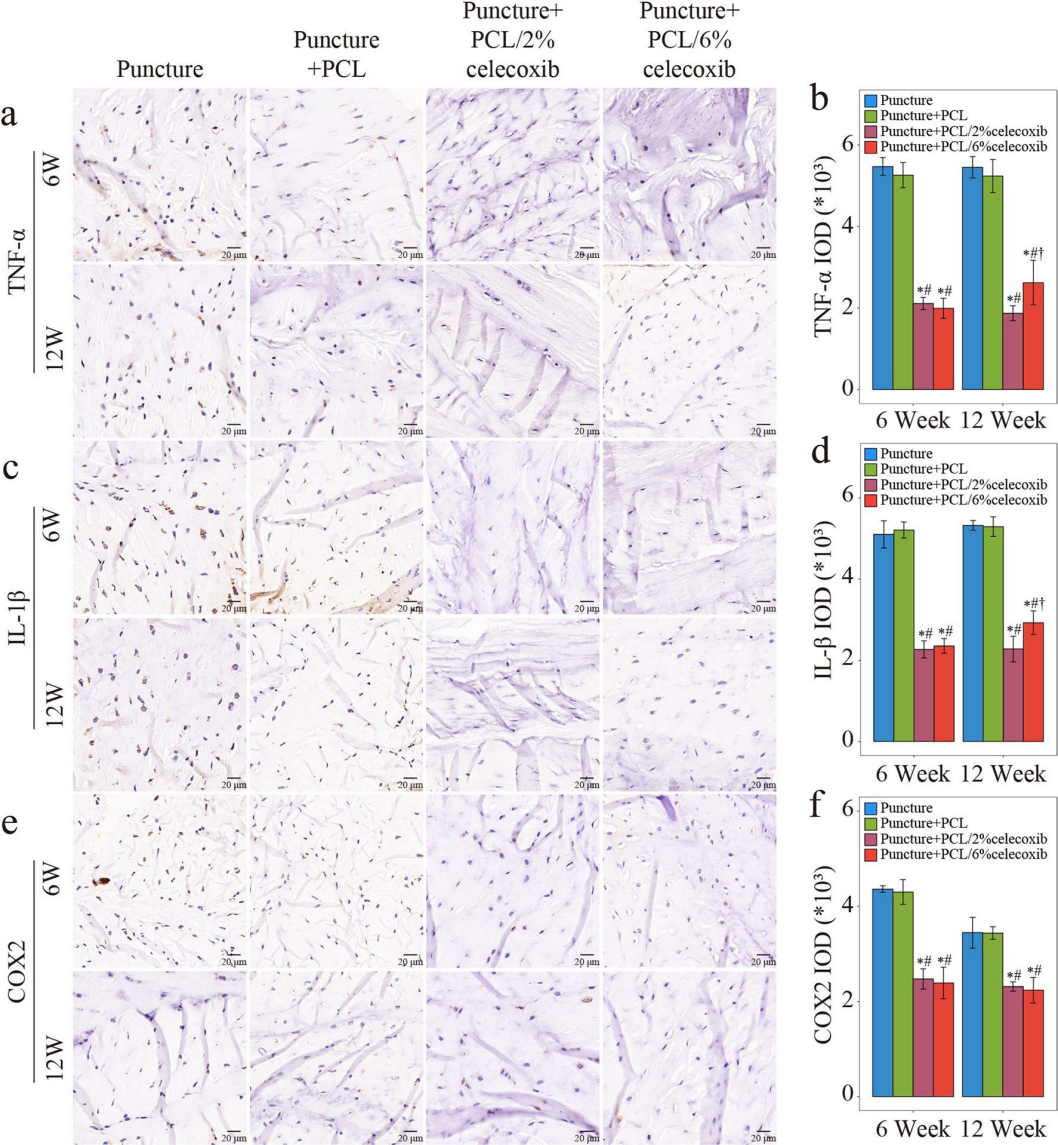
Evaluation of expression of inflammatory cytokines and COX2 with the treatment of PCL, PCL/2% celecoxib nano fibers, and PCL/6% celecoxib nano fibers. **a**–**f** IHC results of TNF-α (**a**, **b**), IL-1β (**c**, **d**), and COX2 (**e**, **f**) in NP of rabbits treated with PCL, PCL/2% celecoxib, and PCL/6% celecoxib nano fibers for 6 weeks and 12 weeks. *P < 0.05 compared with the puncture group, ^#^P < 0.05 compared with the puncture + PCL group, and ^†^P < 0.05 compared with the puncture + PCL/2% celecoxib group

IHC was performed to assess the expression of proinflammatory cytokines and COX2 at week 6 and week 12 postoperatively. TNF-α and IL-1β were the major inflammatory cytokines highly correlated with IDD severity [33]. COX2 played an important role in the inflammation in IDD and was specially inhibited by celecoxib [34, 35]. As shown in Fig. 7a–f, TNF-α, IL-1β, and COX2 were highly expressed in the puncture group and the PCL nano fiber group. Compared with the expression in the puncture group, expressions of TNF-α, IL-1β and COX2 were reduced in the PCL/2% celecoxib nano fiber group and the PCL/6% celecoxib nano fiber group (Fig. 7a–f).

In addition, only at week 12, the expression of TNF-α and IL-1β was slightly higher in the PCL/2% celecoxib nano fiber group than that in the PCL/6% celecoxib nano fiber group, and there was no statistical difference between the two groups at week 6. Meanwhile, the expression of COX2 had no significant difference between the two groups at both week 6 and week 12. Thus, it was presumed that both low-dose celecoxib-loaded PCL nano fibers and PCL/high-dose celecoxib nano fibers could reduce the expression of inflammatory cytokines. Whereas, since low-dose celecoxib-loaded PCL nano fibers were more effective in alleviating IDD under other mechanisms, the relatively severe degeneration led to high expression of inflammatory cytokines in the PCL/high-dose celecoxib nano fiber group.

#### Celecoxib-loaded PCL nano fibers promoting Chsy 3 expression

**Fig. 8:**
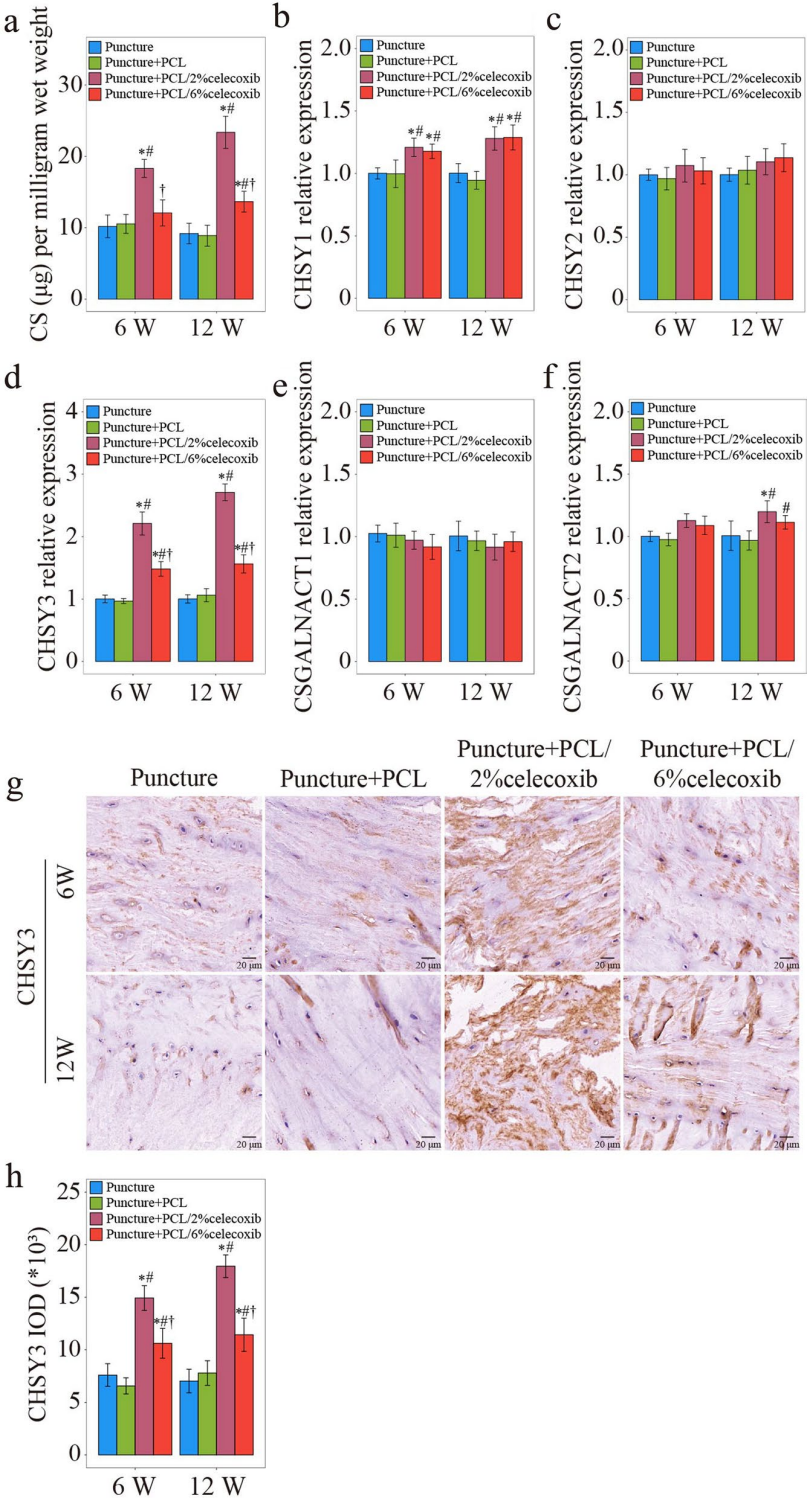
Celecoxib-loaded PCL nano fibers were promoting CHSY3 expression. **a** CS concentration detected by DMMB analysis at week 6 and week 12. **b**–**f** PCR results of CHSY1 (**b**), CHSY2 (**c**), CHSY3 (**d**), CSGALNACT1 (**e**), and CSGALNACT 2 (**f**) in NP of rabbit IDD models treated with PCL, PCL/2% celecoxib, and PCL/6% celecoxib nano fibers for 6 weeks and 12 weeks. **g**, **h** IHC result of CHSY3 in NP of rabbit IDD models treated with PCL, PCL/2% celecoxib, and PCL/6% celecoxib nano fibers for 6 weeks and 12 weeks. *P < 0.05 compared with the puncture group, ^#^P < 0.05 compared with the puncture + PCL group, and ^†^P < 0.05 compared with the puncture + PCL/2% celecoxib group

Our previous studies found a noticeable chondroitin sulfate (CS) reduction in degenerative NP cells [36]. Meanwhile, glycosyltransferases, including chondroitin sulfate synthase 1 (CHSY1), chondroitin sulfate synthase 2 (CHSY2), chondroitin sulfate synthase 3 (CHSY3), chondroitin sulfate *N*-acetylgalactosaminyltransferase 1 (CSGALNACT1), and chondroitin sulfate *N*-acetylgalactosaminyltransferase 2 (CSGALNACT2), were highly correlated with the severity of IDD [36]. Therefore, DMMB assay, RT-PCR, and IHC were performed to detect the expressions of CS and glycosyltransferases. DMMB assay showed that CS concentration was sharply reduced in the puncture group and the PCL group. On the contrary, CS concentration returned to normal in the PCL/celecoxib group (Fig. 8a). For identifying the cause of CS recovery in the PCL/celecoxib group, we used RT-PCR to detect mRNA expression of CHSY1, 2, 3, and that of CSGALNACT1, 2. All the expressions were reduced in the puncture group and the PCL group but were increased in the PCL/2% celecoxib nano fiber group and PCL/6% celecoxib nano fiber group (Fig. 8b–f). Interestingly, compared with other glycosyltransferases, CHSY3 was significantly increased in the PCL/2% celecoxib group and slightly increased in the PCL/6% celecoxib group, although decreased in both the puncture group and the PCL group (Fig. 8d). IHC was performed to further evaluate the expression of CHSY 3 (Fig. 8g, h). The trend of CHSY3 expression was coincident with that detected by PCR. CHSY3 was more expressed in the PCL/2% celecoxib group than in other groups. In conclusion, the findings of the study suggested that CHSY3 expression was significantly higher with low-dose celecoxib and lower with high-dose celecoxib. Low-dose celecoxib might regulate IDD through its effect on CHSY3 expression.

### CHSY3 being indispensable in IDD therapy with low-dose celecoxib

#### CHSY3^−/−^ abolishing the effect of IDD therapy with low-dose celecoxib

**Fig. 9:**
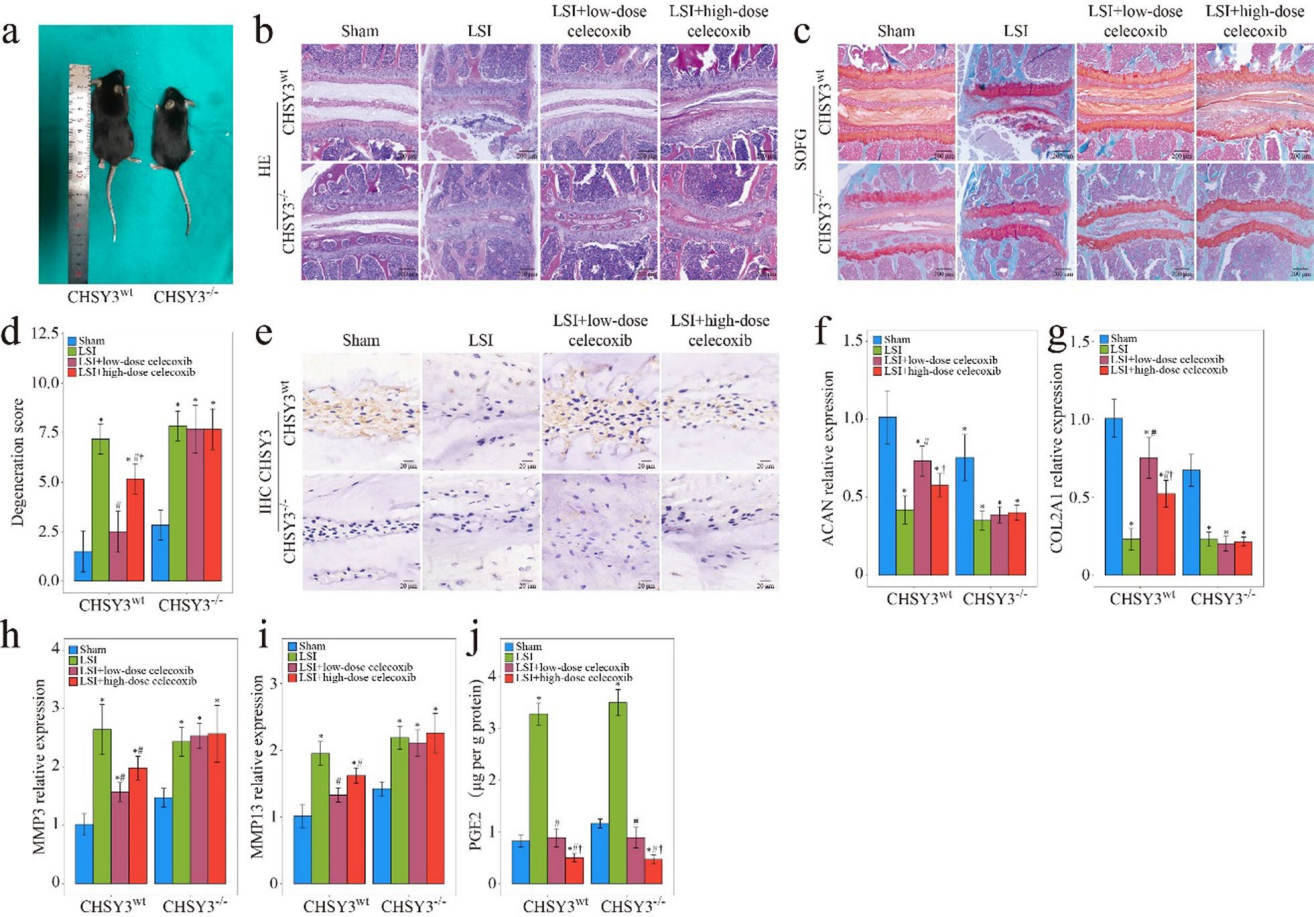
CHSY3^−/−^ abolished the effect of IDD therapy with low-dose celecoxib. **a** HE staining. **b** SOFG staining of the intervertebral disc of WT and CHSY3^−/−^ mice treated with low-dose celecoxib and high-dose celecoxib. **c** WT and CHSY3^−/−^ mice. **d** Degeneration score of HE. **e** IHC result of CHSY3 of the intervertebral disc of WT and CHSY3^−/−^ mice. **f**–**i** PCR results of ACAN (**f**), COL2A1 (**g**), MMP3 (**h**), MMP13 (**i**) of the intervertebral disc of WT and CHSY3^−/−^ mice. **j** PGE2 concentration of the intervertebral disc detected by ELISA. *P < 0.05 compared with the sham group, ^#^P < 0.05 compared with the low LSI group, and ^†^P < 0.05 compared with the high LSI group

Celecoxib at a high dose was effective in relieving pain but had a dose-dependent effect. Low-dose celecoxib resulted in a physiological PGE2 concentration to induce bone formation, while high-dose celecoxib led to a very low concentration of PGE2 and was unfavorable for bone formation [7]. PGE2/EP4-mediated skeletal interoception to mediate bone homeostasis was proved [37]. Bone formation was improved by low-dose celecoxib at a physiological PGE2 concentration may involve activation of skeletal interoception [37]. In this research, the treatment effects of PCL/2% celecoxib fibrous nano fibers for IDD were better than those of the PCL/6% celecoxib group, and PCL/2% celecoxib nano fibers induced a significant increase. To elucidate whether CHSY3 mediated IDD therapy with low-dose celecoxib, we performed experiments on CHSY3^−/−^ mice and CHSY3^wt^ mice in the LSI IDD mouse model (Fig. 9a). Low dose (20 mg kg^−1^ per day) and high dose (80 mg kg^−1^ per day) were administered to the mice with LSI for 1 to 4 weeks.

In the fourth week after the operation, histological and IHC staining were conducted. HE and SOFG staining showed that in punctured CHSY3^wt^ mice, low-dose celecoxib reduced the number of NP cells, causing structural disturbance of the intervertebral disc. However, this effect was abolished in CHSY3^−/−^ mice (Fig. 9b, c). Low-dose celecoxib significantly restored proteoglycan content in CHSY3^wt^ mice after puncture but not in CHSY3^−/−^ mice, as shown in SOFG staining. The degeneration score graph also showed that low-dose celecoxib efficiently reduced the post-puncture degeneration score in CHSY3^wt^ mice but had no effect on the punctured CHSY3^−/−^ mice (Fig. 9d). As shown in Fig. 9e, low-dose celecoxib restored CHSY3 expression in CHSY3^wt^ mice. PCR was performed to evaluate the ECM metabolism of NP, and it was found that only in CHSY3^wt^ mice, low-dose celecoxib could promote the expression of ACAN and COL II, and inhibit MMP3 and MMP13 expression (Fig. 9f–i). Meanwhile, PGE2 in the low-dose group was maintained at the same level as that in the sham group in CHSY3^wt^ mice and CHSY3^−/−^ mice, and physiological PGE2 effectively regulates the activation of skeletal interoception. In conclusion, these results suggested that CHSY3 was indispensable in IDD therapy with low-dose celecoxib. These findings highlighted the potential of CHSY3 as a target for therapeutic intervention in IDD and suggested that low-dose celecoxib might help prevent or treat IDD by targeting CHSY3. By maintaining PGE2 levels at physiological concentrations and promoting CHSY3 expression, low-dose celecoxib might help regulate interoception and prevent IDD.

**Fig. 10:**
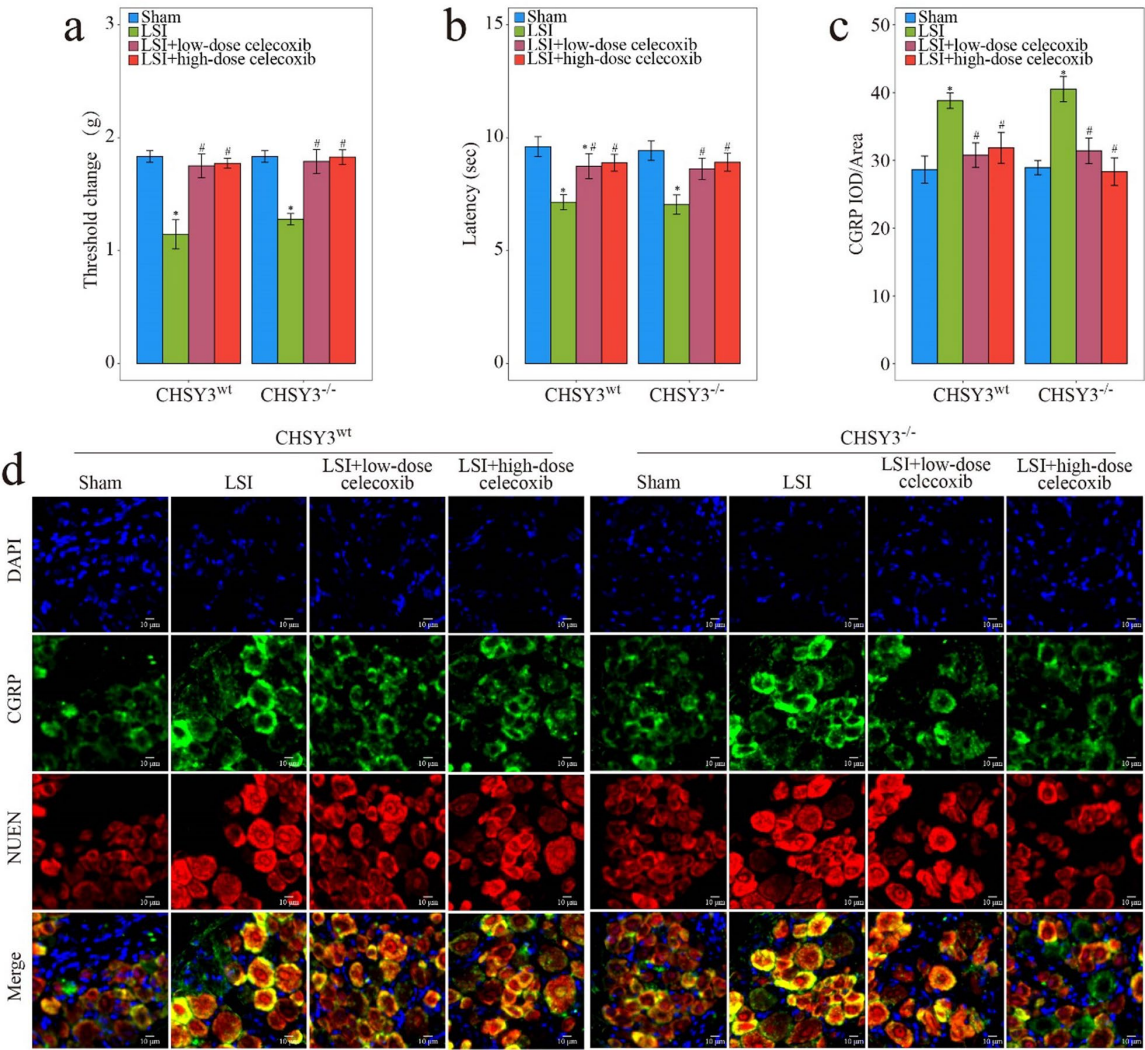
Evaluation of pain condition with low-dose celecoxib and high-dose celecoxib treatment. **a** Von Frey test and **b** Hot plate test of WT and CHSY3^−/−^ mice treated with low-dose celecoxib and high-dose celecoxib for 8 weeks. **c**, **d** IHC analysis of CGRP, NUEN, and DAPI of DRG of WT and CHSY3^−/−^ mice treated with low-dose celecoxib and high-dose celecoxib for 8 weeks. *P < 0.05 compared with the sham group, ^#^P < 0.05 compared with the low LSI group, and ^†^P < 0.05 compared with the high LSI group

Pain assessment was evaluated by the Von Frey test, hot plate test, and IF staining of DRG. In the Von Frey test, low-dose celecoxib restored the mechanical punctate withdrawal threshold values in CHSY3^wt^ and CHSY3^−/−^ mice after puncture (Fig. 10a). The hot plate test showed that under low-dose celecoxib treatment, latencies were decreased in CHSY3^wt^ mice after the puncture, and the same results were found in the punctured CHSY3^−/−^ mice (Fig. 10b). CGRP reportedly transmitted pain signals and induced hyperalgesia [38]. When CHSY3^wt^ mice and CHSY3^−/−^ mice were induced by puncture, CGRP increased and returned to normal physiological levels under the effect of low-dose celecoxib (Fig. 10c, d). It was proved that the regulation of CHSY3 by low-dose celecoxib relieved IDD independent of its analgesic effect.

## Conclusions

In summary, we synthesized low-dose celecoxib-loaded PCL nano fibers by electrostatic spinning technology and demonstrated their ability to alleviate IDD. The effect of low-dose celecoxib-loaded PCL nano fibers on ECM anabolic metabolic improvement in vitro and in vivo through interoception was investigated. More importantly, CHSY3 is indispensable in the treatment of IDD with low-dose celecoxib. Our study shows that low-dose celecoxib-loaded PCL nano fibers will be a promising drug for IDD inhibition.

## Materials and methods

### Preparation of celecoxib-loaded PCL nano fibers

Celecoxib-loaded PCL nano fibers were produced by PCL (Mw = 80 kDa, Sigma-Aldrich, Saint Louis, USA) and celecoxib (Sigma-Aldrich, Saint Louis, USA) according to a previously described method [12]. Firstly, 1 g PCL, 20 mg (0.2% wt/v), or 60 mg celecoxib (0.6% wt/v) was added into 10 mL of 1,1,1,3,3,3-hexafluoro-2-propanol (HFIP, Shanghai Darui Fine Chemical Co., Ltd) solution. Secondly, when the solution propulsion rate was set to 2 mL/h, the mixed solution was infused with a precision syringe pump. Meanwhile, the solution was electrospun with the voltage setting at 15.0 kV and collected by a slow-rotating mandrel placed 15 cm away from the needle tip. Lastly, the obtained celecoxib-loaded PCL nano fibers were dried in a vacuum oven (LGJ-10C, Forging Technology Development (Beijing) Co., Ltd., China) at room temperature for 1 week to ensure complete removal of residual solvent.

### Fabrication of celecoxib-loaded PCL nano fibers

The morphology of celecoxib-loaded PCL nano fibers was investigated by electron microscopy (SEM, FEI Quanta 200, Netherlands), and every sample was observed at least five times. Image J software was used to measure at least 50 fiber diameters in each group, and then the distribution of electrospun fiber diameters was calculated. Fourier transform infrared spectroscopy (FTIR, Thermo Nicolet Co., USA) was used to detect the structural properties of PCL/celecoxib nano fibers, and X-Ray Diffraction (XRD, Brooke Advance D8, Swiss) was used to assess the presence of phases and crystallinity of electrospun nano fibers. Tensile test samples were processed into specimens with a size of 20 mm × 10 mm, and tensile tests were performed on a universal testing machine (Instron 5943, INSTRON, USA). In the celecoxib release experiment, each sample of 100 mg fibrous nano fibers was immersed in 20 mL phosphate-buffered saline (PBS, pH 7.4) with lipase (0.05 mg/mL). The release kinetics of celecoxib were investigated in a thermostatic shaking water bath (37 °C, Taichang Medical Apparatus Co., Jiangsu, China) at the frequency of 100 rpm/min. At scheduled time points, equal parts of 5.0 mL release buffer were taken and refreshed with 5.0 mL PBS. The release of celecoxib was measured by spectrophotometry with a UV-2550 spectrophotometer (Shimadzu, Japan).

### Culture of primary human NP cells

Human NP tissues were collected from a patient undergoing lumbar discectomy and fusion surgery in Shanghai Changzheng hospital, and patient consent forms were obtained before surgery. NP cells were cultured with DMEFM-F12 medium (Gibco, Grand Island, NY, USA), complemented with fetal bovine serum (Gibco, Grand Island, NY, USA) and 1% penicillin–streptomycin antibiotics (Gibco, Grand Island, NY, USA) in an incubator with 5% CO2/95% air at 37 °C. The NP cells culture media was changed every 2 days, and NP cells were passaged at 80% confluence.

### Cell morphology and immunofluorescence staining

Cytoskeleton and nucleus were stained respectively by Alexa Fluor 488 Phalloidin (Abcam, Cambridge, UK) and DAPI (Solarbio, Beijing, China). Samples were fixed with paraformaldehyde for 15 min and then rinsed with PBS three times. 0.2% Triton X-100 was employed to permeabilize for 15 min, and 0.1% bovine serum albumin solution was used to block samples for 30 min. At last, samples were incubated with Alexa Fluor 488 Phalloidin and DAPI for 2 h and were detected by an inversion fluorescence microscope.

### Cell counting kit-8 (CCK-8) assay

NP cells were seeded in a 96-well plate and were incubated with CCK-8 solution (Dojindo, Kumamoto, Japan). After 2 h, the solution was measured at 450 nm.

### Establishment of an IDD rabbit model

A fine needle puncture method was used to build an IDD rabbit model. Rabbits (n = 20) were anesthetized with pentobarbitone sodium (25 mg/kg^−1^) via intravenous injection in the ear vein, and intervertebral discs were exposed through a lateral retroperitoneal approach. L2/3, L3/4, and L4/5 intervertebral discs were punctured with an 18-gauge needle, then the annulus fibrosus was inserted 5 m and finally rotated 360° to ensure adequate disc injury. Nano fibers were fixed to the lesions of intervertebral discs.

### Establishment of a lumbar spine instability (LSI) mouse model

Eight-week–old CHSY3^−/−^ mice and WT mice were used for the LSI model. Mice were anesthetized with an intraperitoneal injection of pentobarbital (40 mg/kg), and lumbar spines were exposed via a posterior approach. L2–L5 spinous processes and articular processes were resected to induce lumbar spine instability.

### Immunocytochemistry and immunofluorescence analyses

NP tissues were fixed with paraformaldehyde for 48 h and decalcified with ethylenediaminetetraacetic acid for 3 weeks. The samples were embedded in paraffin and were cut at 4 μM thickness. The sections were incubated with primary antibodies MMP3 (ab234405, Abcam, MA, USA), MMP3 (66338-1-lg, proteintech, Wuhan, China), MMP13 (ab219620, Abcam, MA, USA), MMP13 (ab1010, Abcam, MA, USA), ACAN (ab186414, Abcam, MA, USA), ACAN (ab3778, Abcam, MA, USA), Col II (ab34712, Abcam, MA, USA), Col II (ab185430, Abcam, MA, USA), CHSY3 (PL0306862, PLLABS, Richmond, BC, Canada), TNF-α (6029-1-lg, proteintech, Wuhan, China), IL-1β (66737-1-lg, proteintech, Wuhan, China), COX2 (66351-1-lg, proteintech, Wuhan, China). Incubation was then performed with second antibodies. DAPI was used to stain the nucleus and the slides were sealed with anti-fluorescence quenching sealing tablets.

### LC–MS/MS

Celecoxib was measured by LC–MS/MS with a QTRAP 5500 triple-quadruple mass spectrometer (SCIEX, Framingham, MA) in positive electrospray ionization mode by multiple reaction monitoring data acquisition with an Agilent 1200 HPLC (Agilent Technologies, Santa Clara, CA). Chromatography was performed by automated injection on a Kinetex Biphenyl column, 50 × 2.1 mm, 2.6 μm particle size (Phenomenex, Torrance, CA). HPLC flow was maintained at 400 μL/min with mobile phases of A = 0.1% formic acid in water and B = 0.1% formic acid in acetonitrile. The initial conditions were 50% A, the gradient was ramped to 5% A at 2.5 min, and then returned to 50% A immediately. The total run time was 7 min. Data acquisition and quantification were performed in Analyst 1.6.2 software (Sciex).

### Real-time PCR

According to the manufacturer’s instructions, TRIzol reagent (Takara bio, Japan) was performed to extract total RNA from NP tissues and cells, and total RNA was transcribed into complementary DNA (cDNA) with a real-time reverse transcriptase-polymerase chain reaction kit (Takara Bio, Japan). SYBR Green PCR Master Mix (Takara Bio, Japan) was used to conduct RT-PCR reaction on an applied biosystems (ABI) QuantStudio 6 Flex (Thermo Fisher Scientific, USA). Samples were performed three times independently for each RT-PCR, and the primer sequences for each gene were shown in Table 1. With the 2− ΔΔCt method, the relative expression of target mRNAs was calculated after normalization with GAPDH.

**Table 1:** Primer sequences used for real-time PCR

Target	Forward primers, 5′–3′	Reverse primers, 5′–3′
Human		
MMP 3	GACACCAGCATGAACCTTGTT	GGAACCGAGTCAGGTCTGTG
MMP 13	ATTAAGGAGCATGGCGACTTCT	GCCCAGGAGGAAAAGCATGA
ACAN	GGTCTCACTGCCCAACTACC	CACGATGCCTTTCACCACGA
COL II	GATGGCTGCACGAAACATACC	GCCCTATGTCCACACCGAAT
GAPDH	GCACCGTCAAGGCTGAGAAC	TGGTGAAGACGCCAGTGGA
Rabbit		
GAPDH	TCACCATCTTCCAGGAGCGA	CACAATGCCGAAGTGGTCGT
CHSY1	ATGATGCTCAAGTACATGCACGA	TCAGGCTGTCCTCCAAGAGC
CHSY2	ATGCGGGCATCCCTGCTG	TCAGGTGCTGTGGCCCTG
CHSY3	ATGGCCGTGCGCTCCCGC	TCAGGAGAGAGTTCGATTGTACCTG
CSGALNACT1	ATGATGGTCCGCAGGGGG	TCATGTCTTCTTGCTGCCCG
CSGALNACT2	ATGCCTAGAAGAGGGCTGGC	CTAACCAGCCGCCTCGCT
GAPDH	ATGGTGAAGGTCGGAGTGAACG	TTACTCCTTGGAGGCCATGTG
Mouse		
MMP-3	ATGAAAATGAAGGGTCTTCCGG	GCAGAAGCTCCATACCAGCA
MMP 13	TGATGATGAAACCTGGACAAGCA	GGTCCTTGGAGTGATCCAGACCTA
ACAN	AAACCTGGCGTGAGAACTGT	CCACTGACACACCTCGGAAG
COL II	CCAGATTGAGAGCATCCGCA	ACTTTCATGGCGTCCAAGGT
GAPDH	AGGTCGGTGTGAACGGATTTG	TGTAGACCATGTAGTTGAGGTCA

### Enzyme-linked immunosorbent assay (ELISA)

NP cells were seeded in a 96-well plate and supernatants were collected. After PBS (1X) was added to NP tissues, mixtures were homogenized for 30 s and centrifuged for 10 min for our collection of the supernatants. Standard wells and sample wells were set on a 96-well plate, and according to the manufacturer’s instructions, the concentration of PGE2 in the supernatants was detected with PGE2 ELISA Kit (Multi Sciences, Hangzhou, China).

### Von Frey test

Von Frey test was conducted according to the methods of Vuong et al. [39]. Briefly, before the test day, animals were individually placed in test chambers for 10 min to ensure two consecutive days of acclimation. The plantar surface of the hind paw was stimulated with Von Frey filaments. The next weaker stimulus was considered the withdrawal threshold when a withdrawal response occurred. The 50% paw withdrawal threshold was calculated according to the method described by Chaplan et al. [40].

### Hot plate test

The hot plate test was performed to measure the somatic pain response [28]. On the testing day, animals were placed on an advanced hot plate and acclimated for 30 s. The hot plate was heated to 52 ℃, and animals were immediately removed when they showed a nociceptive response.

### Statistical analysis

All statistical analyses were performed in Prism software (GraphPad 8). All experimental data were analyzed with ANOVAs or t-tests as data were determined the normality with the Shapiro–Wilk test and were followed by Sidak’s, Tukey’s, and Dunnett’s post hoc multiple comparison test. Significance levels were indicated as follows: *: P < 0.05, #: P < 0.05, †: P < 0.05. All values were shown as mean ± standard error of the mean (SEM), and n represented the number of animals or cells examined.

## Supplementary Information

**Additional file 1:** **Fig. S1a.** Cumulative release curve of low-dose celecoxib and high-dose celecoxib.

## Data Availability

The data that support the findings of this study are available from the corresponding author upon reasonable request.
