# Supplementary material for: Low-dose celecoxib-loaded PCL fibers reverse intervertebral disc degeneration by up-regulating CHSY3 expression
Source: J Nanobiotechnology. 2023 Mar 3;21:76. doi: 10.1186/s12951-023-01823-4 (PMC9983215; doi:10.1186/s12951-023-01823-4)
Supplement: Supplementary file 1 — Additional file 1: Fig. S1a. Cumulative release curve of low-dose celecoxib and high-dose celecoxib. [file 12951_2023_1823_MOESM1_ESM.pdf]

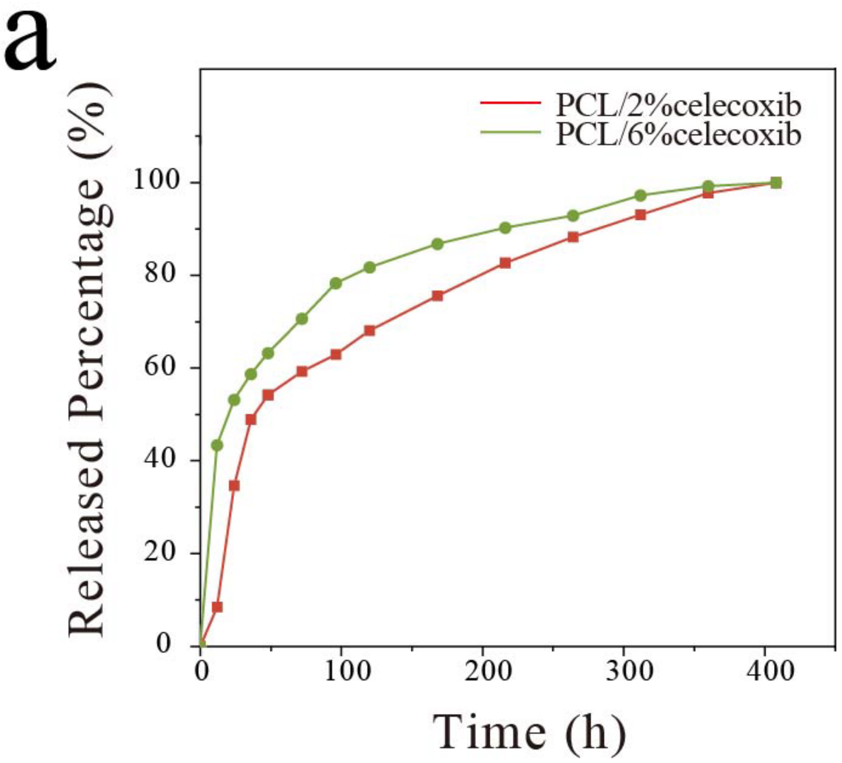


Figure supplement 1a cumulative release curve of low-dose celecoxib and high-dose celecoxib

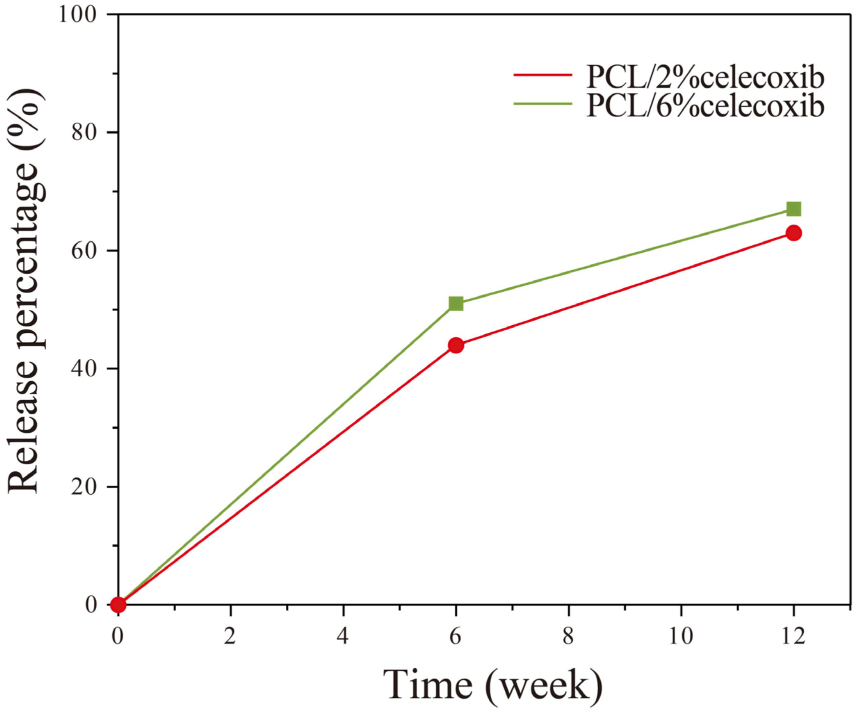


Figure supplement 2 the release efficiency of celecoxib from PCLcelecoxib fibers

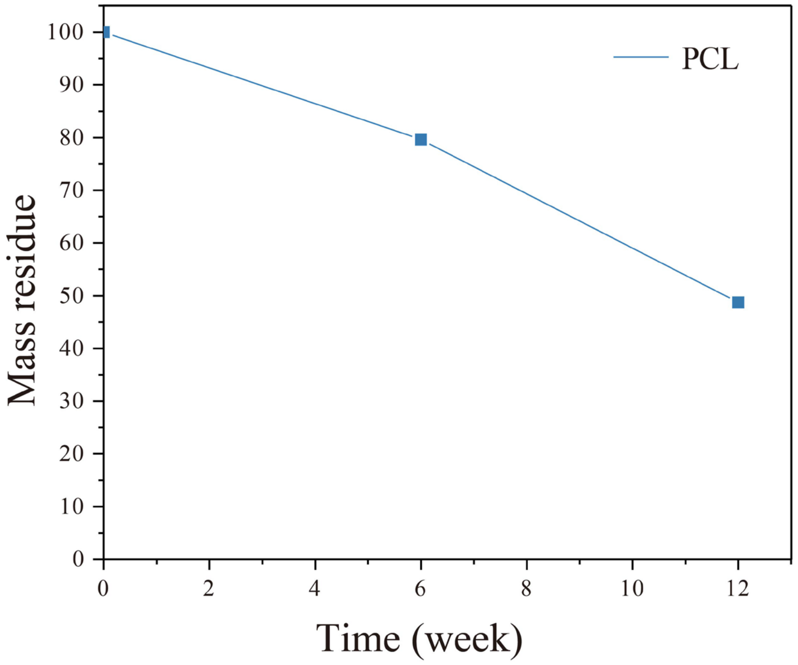

Figure supplement 3 the degradation rate and biosafety of PCL nanofibers
